# Automated hepatic steatosis assessment on dual-energy CT-derived virtual non-contrast images through fully-automated 3D organ segmentation

**DOI:** 10.1007/s11547-024-01833-8

**Published:** 2024-06-13

**Authors:** Sun Kyung Jeon, Ijin Joo, Junghoan Park, Jeongin Yoo

**Affiliations:** 1 Department of Radiology, Seoul National University Hospital and Seoul National University College of Medicine, 101 Daehak-ro, Jongno-gu, Seoul, 03080 Korea; 2 Department of Radiology, Seoul National University College of Medicine, Seoul, South Korea; 3 Institute of Radiation Medicine, Seoul National University Medical Research Center Seoul National University Hospital, Seoul, Korea

**Keywords:** Hepatic steatosis, Deep learning, Segmentation, Volumetry, Dual-energy computed tomography

## Abstract

**Purpose:**

To evaluate the efficacy of volumetric CT attenuation-based parameters obtained through automated 3D organ segmentation on virtual non-contrast (VNC) images from dual-energy CT (DECT) for assessing hepatic steatosis.

**Materials and methods:**

This retrospective study included living liver donor candidates having liver DECT and MRI-determined proton density fat fraction (PDFF) assessments. Employing a 3D deep learning algorithm, the liver and spleen were automatically segmented from VNC images (derived from contrast-enhanced DECT scans) and true non-contrast (TNC) images, respectively. Mean volumetric CT attenuation values of each segmented liver (L) and spleen (S) were measured, allowing for liver attenuation index (LAI) calculation, defined as L minus S. Agreements of VNC and TNC parameters for hepatic steatosis, i.e., L and LAI, were assessed using intraclass correlation coefficients (ICC). Correlations between VNC parameters and MRI-PDFF values were assessed using the Pearson’s correlation coefficient. Their performance to identify MRI-PDFF ≥ 5% and ≥ 10% was evaluated using receiver operating characteristic (ROC) curve analysis.

**Results:**

Of 252 participants, 56 (22.2%) and 16 (6.3%) had hepatic steatosis with MRI-PDFF ≥ 5% and ≥ 10%, respectively. L_VNC_ and LAI_VNC_ showed excellent agreement with L_TNC_ and LAI_TNC_ (ICC = 0.957 and 0.968) and significant correlations with MRI-PDFF values (r = − 0.585 and − 0.588, Ps < 0.001). L_VNC_ and LAI_VNC_ exhibited areas under the ROC curve of 0.795 and 0.806 for MRI-PDFF ≥ 5%; and 0.916 and 0.932, for MRI-PDFF ≥ 10%, respectively.

**Conclusion:**

Volumetric CT attenuation-based parameters from VNC images generated by DECT, via automated 3D segmentation of the liver and spleen, have potential for opportunistic hepatic steatosis screening, as an alternative to TNC images.

**Supplementary Information:**

The online version contains supplementary material available at 10.1007/s11547-024-01833-8.

## Introduction

Hepatic steatosis, characterized by excessive fat accumulation in hepatocytes, is a key histological feature of steatotic liver diseases, including metabolic dysfunction-associated steatotic liver disease and alcohol-associated liver disease [1, 2]. Simple steatosis can progress to steatohepatitis and cirrhosis and is associated with the development of cardiovascular diseases or diabetes [3]. Moreover, early intervention in hepatic steatosis can halt or reverse disease progression, underscoring the significance of early detection and accurate assessment [4]. Although liver biopsy has traditionally been regarded as the gold standard for diagnosing hepatic steatosis, its invasive nature and notable inter-reader variability have prompted a substantial clinical demand for non-invasive methods [5]. Recently, chemical shift-encoded MRI proton density fat fraction (MRI-PDFF) has gained broad acceptance as a non-invasive reference standard for liver fat quantification, providing accurate and reliable measurements [6, 7]. However, it has drawbacks, including limited accessibility and high costs. Although CT has not been considered a primary diagnostic tool for hepatic steatosis due to its limited sensitivity for mild cases and radiation exposure concerns, its widespread use in various clinical conditions and acceptable performance for moderate or severe steatosis suggest its viability for opportunistic screening [8].

Recent advancements in automated 3D organ segmentation in medical imaging have expanded the feasibility of utilizing volumetric data, encompassing organ volume measurements and automated quantification of radiomic features representing the entire organ [9, 10]. When assessing hepatic steatosis using CT, traditional methods involve time-consuming and labor-intensive manual 2D region of interest (ROI)-based measurements of CT attenuation values. However, with the implementation of deep learning-based 3D segmentation, volumetric CT attenuation can be acquired automatically, and studies have been actively exploring the application of this approach for CT-based steatosis screening in large populations [11, 12].

Dual-energy CT (DECT) is becoming increasingly prominent in clinical practice due to its advantages such as material decomposition and the generation of energy-selective images [13]. A notable application involves providing virtual non-contrast (VNC) images through material decomposition [14]. Specifically, by subtracting iodine content from contrast-enhanced CT images, VNC images can be obtained, offering non-contrast information without additional radiation exposure for true non-contrast (TNC) image acquisition. While previous evaluations of VNC have mainly focused on qualitative aspects, such as stone or calcification detection, as a potential substitute for TNC imaging [15], recent studies have highlighted its role in quantification. These studies demonstrated excellent agreement between VNC and TNC measurements in the CT attenuation values of abdominal organs [16, 17]. Although current CT-based hepatic steatosis assessments typically rely on TNC images, the potential use of VNC images could provide benefits by allowing evaluation even in individuals undergoing contrast-enhanced DECT without TNC. Evaluations in this context have been conducted using 2D ROI methods [17]; however, to our knowledge, no known investigation into the volumetric analysis of VNC images exists.

Therefore, this study aims to evaluate the efficacy of volumetric CT attenuation-based parameters obtained through automated 3D organ segmentation on VNC images from DECT for assessing hepatic steatosis, comparing them to TNC parameters and using MRI-PDFF as the reference standard.

## Materials and methods

This retrospective study received approval from our institution's institutional review board, and the requirement for informed consent was waived due to the study’s retrospective nature.

### Study population

**Fig. 1:**
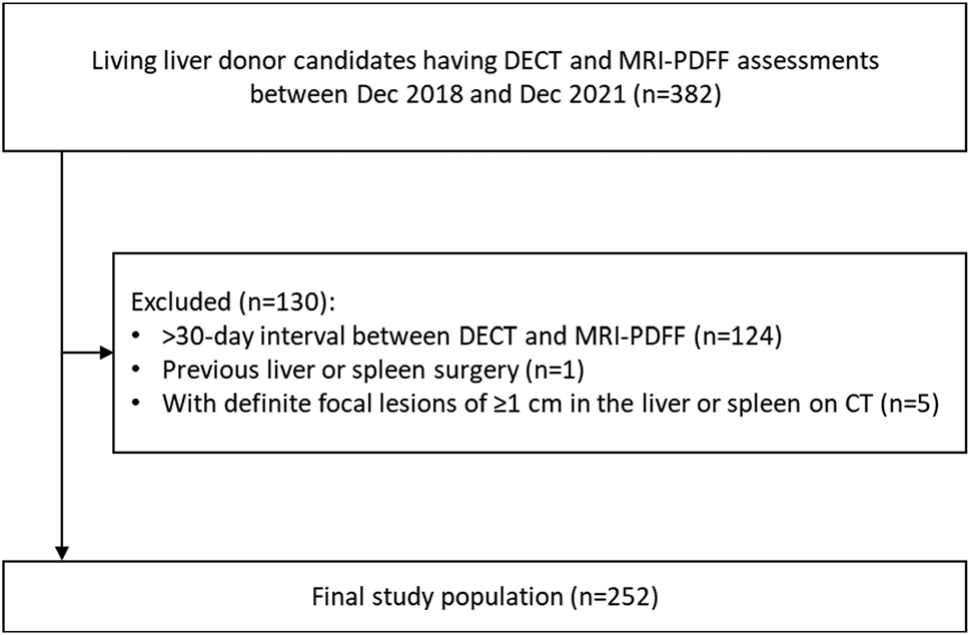
Flowchart of the study population. DECT = dual-energy CT; MRI-PDFF = magnetic resonance imaging-proton density fat fraction

Living liver donor candidates aged ≥ 18 years, who underwent preoperative work-up in our institution between December 2018 and December 2021, were identified through a computerized search of the picture archiving and communication system and electronic medical records. Liver donor candidates had undergone liver CT scans using a dual-source DECT scanner and liver MRI with MRI-PDFF assessment as part of their routine preoperative evaluation. We excluded the following: patients with > 30-day interval between DECT and MRI-PDFF, a history of previous liver or spleen surgery, and definite focal lesions of 1 cm or larger in the liver or spleen on CT (Fig. 1).

### Acquisition of DECT and generation of VNC images

During the study period, preoperative liver CT scans for living liver donor candidates were routinely performed using a dual-source DECT scanner (SOMATOM Force; Siemens Healthineers, Erlangen, Germany). These scans included TNC, arterial, portal venous, and delayed-phase imaging. Post-contrast phases were acquired in the DECT mode at 80 and 150 kVp, while TNC images were obtained in the single-energy CT mode at 120 kVp. Contrast was administered using iobitridol (Xenetix 350; Guerbet) at 520 mg/kg body weight, followed by a saline flush. Detailed CT protocols are described in Supplementary 1.

VNC images were generated from portal venous phase DECT data, acquired 70 s post-contrast administration, using a dedicated post-processing system (Syngo.via; Siemens Healthineers) at the CT console, reconstructed with 2 mm slice thickness and a 1 mm reconstruction interval.

### CT attenuation measurements

#### Automated volumetric measurements

**Fig. 2:**
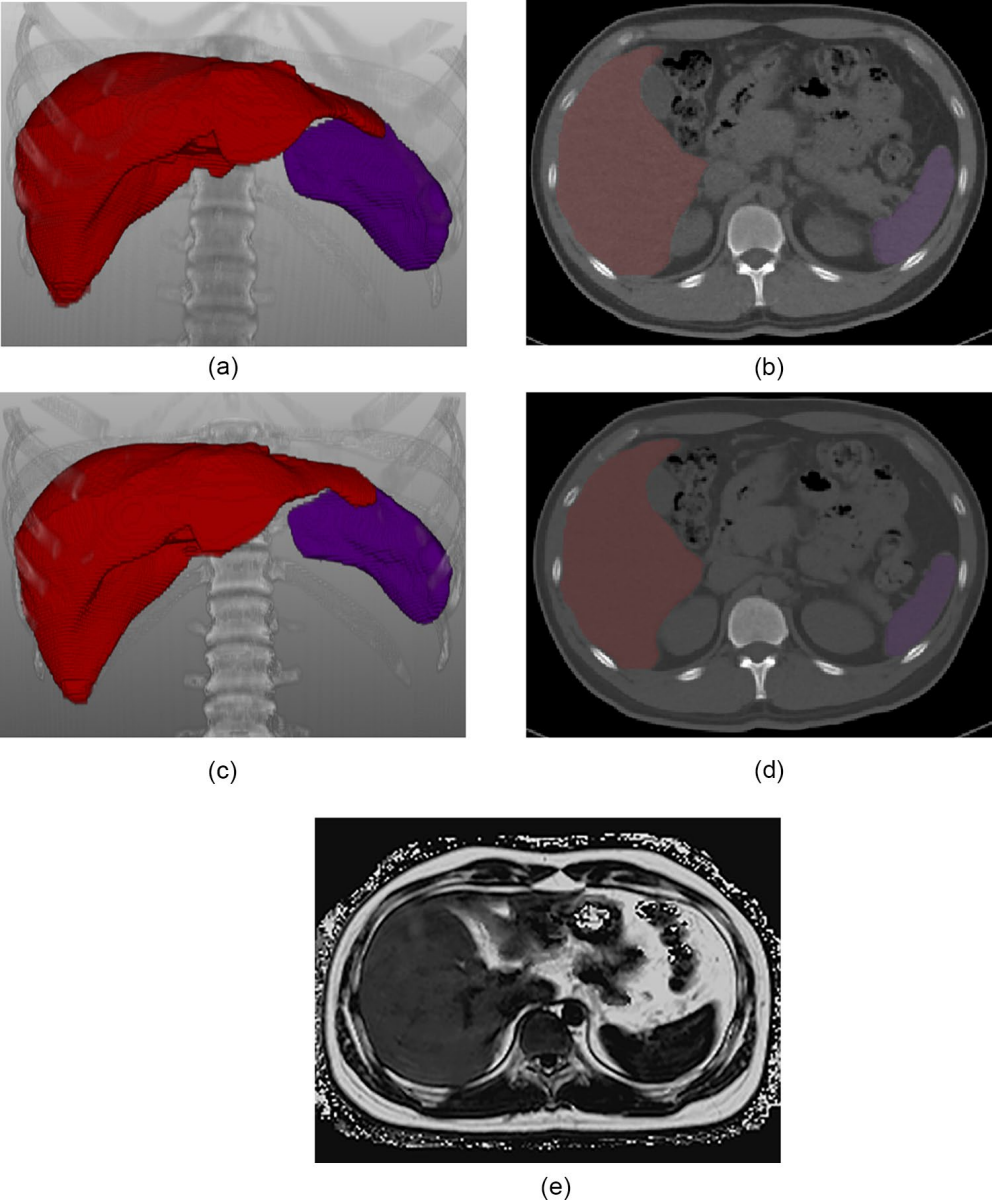
An illustration of volumetric CT attenuation measurement of the liver and spleen in a 27-year-old man with hepatic steatosis. Fully-automated organ segmentation was performed for the liver and spleen on virtual non-contrast (VNC) CT scan (**a**: 3D volume rendering image, **b**: axial image) and true non-contrast (TNC) CT scan (**c**: 3D volume rendering image, **d**: axial image), respectively. Volumetric measurements of CT attenuation values were as follows: L_VNC_ = 48.3 HU, S_VNC_ = 47.0 HU in VNC imaging; L_TNC_ = 44.4 HU, S_TNC_ = 49.2 HU in TNC imaging. Accordingly, LAI_VNC_ was 1.3 HU and LAI_TNC_ was -4.8 HU. Hepatic steatosis was confirmed with an MRI-PDFF value of 14.8% (**e**: MRI-PDFF map). MRI-PDFF = magnetic resonance imaging-proton density fat fraction; L = volumetric mean CT attenuation values of the liver; S = volumetric mean CT attenuation values of the spleen; LAI = liver attenuation index, defined as L minus S

For VNC and TNC imaging, the liver and spleen were segmented automatically using a commercially available deep-learning-based multi-organ segmentation software (DeepCatch v1.2.0.0, MEDICALIP Co. Ltd., Seoul, Korea) (Fig. 2). Its segmentation performance was reported to be dice scores > 0.95 for both the liver and spleen in each of VNC and TNC imaging. After generating a 3D organ mask, the software automatically calculated the mean volumetric CT attenuation of the segmented organs. Mean CT attenuation values (Hounsfield units, HU) of the liver and spleen on VNC imaging were denoted as L_VNC_ and S_VNC_, respectively, and those on TNC imaging were denoted as L_TNC_ and S_TNC_, respectively.

#### Manual 2D ROI-based measurements

For comparison with the volumetric measurements, CT attenuation of the liver and spleen was measured manually using the 2D ROI-based methods on the VNC images. Three circular ROIs were placed in the liver (one in the right anterior section, one in the right posterior section, and one in the left lobe), carefully avoiding vessels and liver edges [18]. Additionally, one ROI was placed in the spleen [19, 20]. The ROIs had a size ranging from 250 to 300 mm^2^, and the size of the four ROIs within each subject was the same. To determine the mean liver attenuation, the values from the three liver ROIs were averaged. To assess the reproducibility of 2D ROI-based methods, measurements were conducted in two separate sessions. The first measurement was used as the representative value for ROI-based measurements, and the results from the second session were utilized solely for assessing reproducibility.

#### CT attenuation-based parameters for assessing hepatic steatosis

We employed the mean liver HU (L_VNC_ and L_TNC_) and the liver attenuation index (LAI) as CT attenuation-based parameters for assessing hepatic steatosis, relying on previous studies that evaluated the utility of CT for this purpose [11, 21–23]. Regarding the LAI, it was calculated as the mean liver HU minus the spleen HU (denoted as LAI_VNC_ and LAI_TNC_ for VNC and TNC images, respectively). The LAI has been reported to be robust across various scan settings using the spleen as an internal reference [21, 22].

#### MRI-PDFF acquisition and measurements

Chemical-shift-encoded MRI-PDFF examinations were conducted using a 3-T MRI scanner (MAGNETOM Skyra; Siemens Healthineers, Erlangen, Germany), with complex-based chemical shift-encoded water-fat reconstruction techniques, acquiring six 2D gradient-echo images. The acquisition parameters included a repetition time (TR) of 9.3 ms, an echo time (TE) of 1.3 ms, an imaging matrix of 256 × 192, a low flip angle (4°), and a 3 mm slice thickness. After that, the PDFF maps were automatically generated using the vendor specific algorithm, incorporating T2* correction calculated from single decay and a multi-peak fat model. MRI-PDFF values, widely accepted as a non-invasive reference standard for liver fat content [6], were determined using the ROI method on the PDFF map as follows: three ROIs with a size of 250–300 mm^2^ were strategically placed in the right anterior section, right posterior section, and left liver [18], while avoiding large vessels, bile ducts, liver edges, and artifacts [1, 24]. The mean value of the three ROIs was then calculated and utilized as the representative PDFF value for each participant.

### Statistical analysis

Regarding the CT attenuation values of the liver and spleen, agreements were assessed between volumetric and manual 2D ROI-based VNC measurements as well as between volumetric VNC and TNC measurements using both the intraclass correlation coefficient (ICC) and Bland–Altman analysis. Additionally, the reproducibility of manual 2D ROI-based VNC measurements across two separate sessions was assessed using ICC. The ICC values were interpreted as follows: ≥ 0.90 indicating excellent; ≥ 0.75 – < 0.90, good; ≥ 0.50 – < 0.75, moderate; and < 0.50, poor [25].

Volumetric VNC parameters for hepatic steatosis (L_VNC_ and LAI_VNC_) were correlated with the corresponding TNC parameters (L_TNC_ and LAI_TNC_) and MRI-PDFF values using Pearson’s correlation coefficient (r). Correlation strength was interpreted as follows:$\left|\text{r}\right|$≥ 0.7, strong; ≥ 0.4 – < 0.7, moderate; ≥ 0.2 – < 0.4, weak; and ≥ 0 – < 0.2, minimal [26]. The diagnostic performances of volumetric VNC parameters for hepatic steatosis (L_VNC_ and LAI_VNC_) were evaluated to identify MRI-PDFF ≥ 5% (indicating mild steatosis) and ≥ 10% (indicating moderate-to-severe steatosis) through receiver operating characteristic (ROC) analysis. Cutoff values for VNC parameters corresponding to MRI-PDFF ≥ 5% and ≥ 10% were determined to maximize the Youden index, achieving > 80% sensitivity, and > 80% specificity, respectively. The corresponding sensitivity and specificity values were then computed. The areas under the ROC curve (AUCs) were compared between the VNC parameters (L_VNC_ vs. LAI_VNC_) and between the VNC and TNC parameters (L_VNC_ vs. L_TNC_ and LAI_VNC_ vs. LAI_TNC_) using the DeLong test.

All statistical analyses were performed using MedCalc version 19.4.0 (MedCalc Software, Ostend, Belgium), with a significance threshold set at *P* < 0.05.

## Results

### Study population

A total of 252 living liver donor candidates, comprising 139 men and 113 women, with a mean age of 37.3 years (range, 18‒64 years), were included in the analysis. Their mean (± standard deviation) MRI-PDFF value was 4.0% (± 3.1) with a range of 0.5‒23.8%. Within this population, 56 patients (22.2%) had hepatic steatosis (MRI-PDFF ≥ 5%), including 16 with moderate-to-severe hepatic steatosis (MRI-PDFF ≥ 10%).

### CT attenuation values of the liver and spleen

#### Volumetric versus 2D ROI-based measurements on VNC imaging

For L_VNC_, the volumetric measurements demonstrated excellent agreement with 2D ROI-based measurements, showing an ICC of 0.979. Regarding S_VNC_, the volumetric measurements showed good agreement with 2D ROI-based measurements, displaying an ICC of 0.862 (Table 1). Mean bias (95% limits of agreement) in volumetric measurements compared to ROI-based measurements was − 5.1 HU (− 8.7 HU to − 1.5 HU) for L_VNC_ and − 5.6 HU (− 10.5 HU to − 0.6 HU) for S_VNC_, respectively (Supplementary Fig. 1). The reproducibility of 2D ROI-based measurements was excellent for L_VNC_ (ICC = 0.982 [95% CI 0.977–0.986]) and good for S_VNC_ (ICC = 0.895 [95% CI 0.865–0.918]).

**Table 1:** Intraclass correlation coefficients between CT attenuation measurement methods

CT attenuation measurement methods	Intraclass correlation coefficient (95% CI)	
	Liver	Spleen
Volumetric versus 2D ROI-based measurements on VNC imaging	0.979 (0.973‒0.984)	0.862 (0.824‒0.893)
VNC versus TNC imaging for volumetric measurements	0.957 (0.945‒0.966)	0.809 (0.755‒0.851)

*ROI* region of interest, *VNC* virtual non-contrast, *TNC* true non-contrast, *CI* confidence interval

#### Volumetric measurements on VNC imaging versus TNC imaging

Volumetric L_VNC_ showed excellent agreement with L_TNC_, having an ICC of 0.957, whereas volumetric S_VNC_ showed good agreement with S_TNC_, presenting an ICC of 0.809 (Table 1). Mean bias (95% limits of agreement) was 1.0 HU (− 3.8–5.7 HU) for L_VNC_ and L_TNC_, and − 4.2 HU (− 9.3–0.8 HU) for S_VNC_ and S_TNC,_ respectively (Supplementary Fig. 2).

### Volumetric VNC parameters for hepatic steatosis

#### Correlation with TNC parameter for hepatic steatosis

**Fig. 3:**
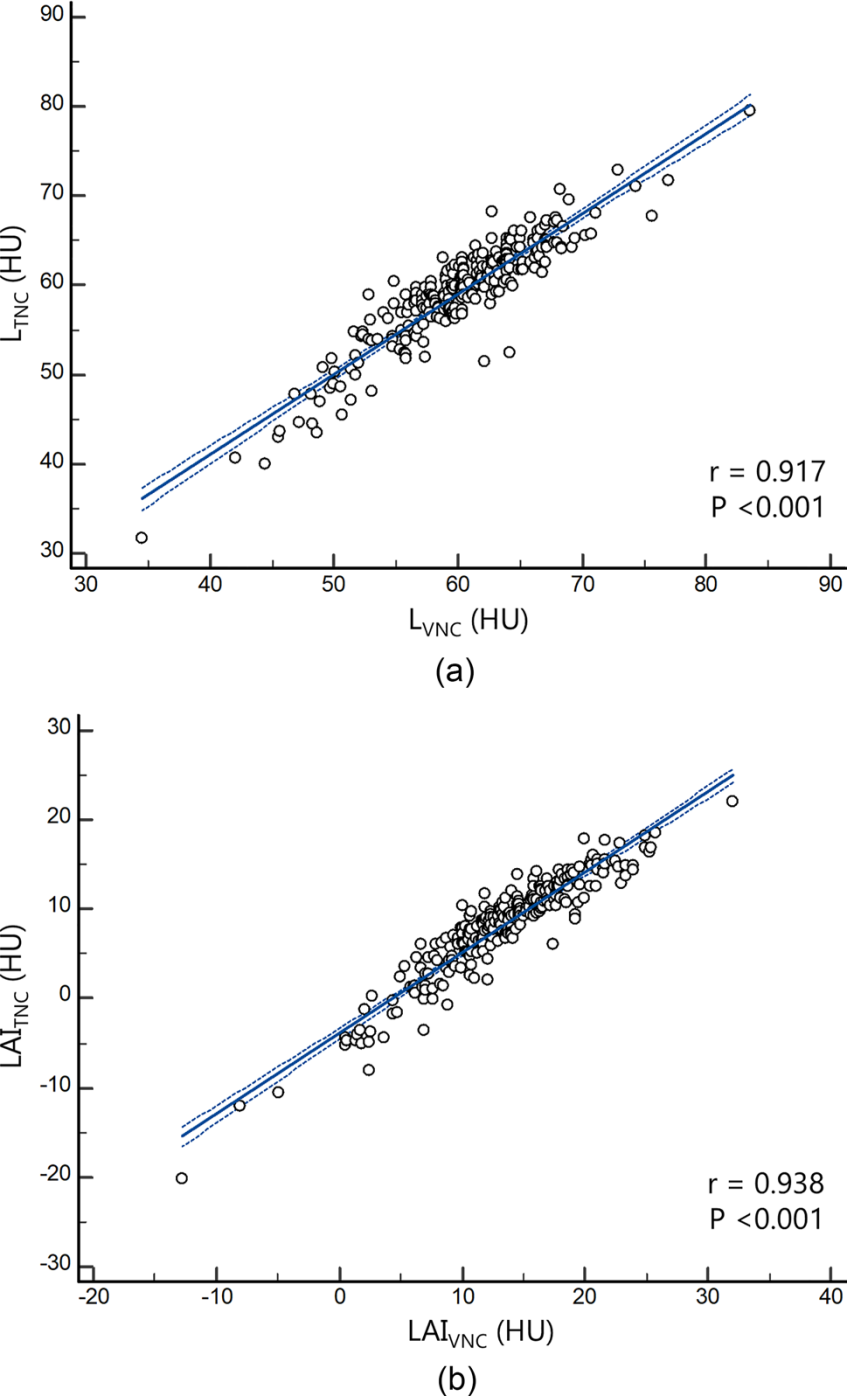
Scatter plots displaying the regression line (solid) and 95% confidence interval (dotted) of virtual non-contrast (VNC) parameters for hepatic steatosis (a: L_VNC_ and b: LAI_VNC_) with true non-contrast (TNC) parameters (a: L_TNC_ and b: LAI_TNC_). ‘r’ denotes Pearson’s correlation coefficient. L = volumetric mean CT attenuation values of the liver; S = volumetric mean CT attenuation values of the spleen; LAI = liver attenuation index, defined as L minus S

VNC parameters for hepatic steatosis, i.e., L_VNC_ and LAI_VNC_, showed strong positive correlations with corresponding TNC parameters (L_TNC_ and LAI_TNC_) (r = 0.917 [95% CI 0.895‒0.935] and 0.938 [95% CI 0.923‒0.952], respectively; Ps < 0.001) (Fig. 3). L_VNC_ and LAI_VNC_ showed excellent agreements with L_TNC_ and LAI_TNC_ (ICCs, 0.957 [95% CI 0.945‒0.966] and 0.968 [95% CI 0.959‒0.975], respectively).

#### Correlation with MRI-PDFF values

**Fig. 4:**
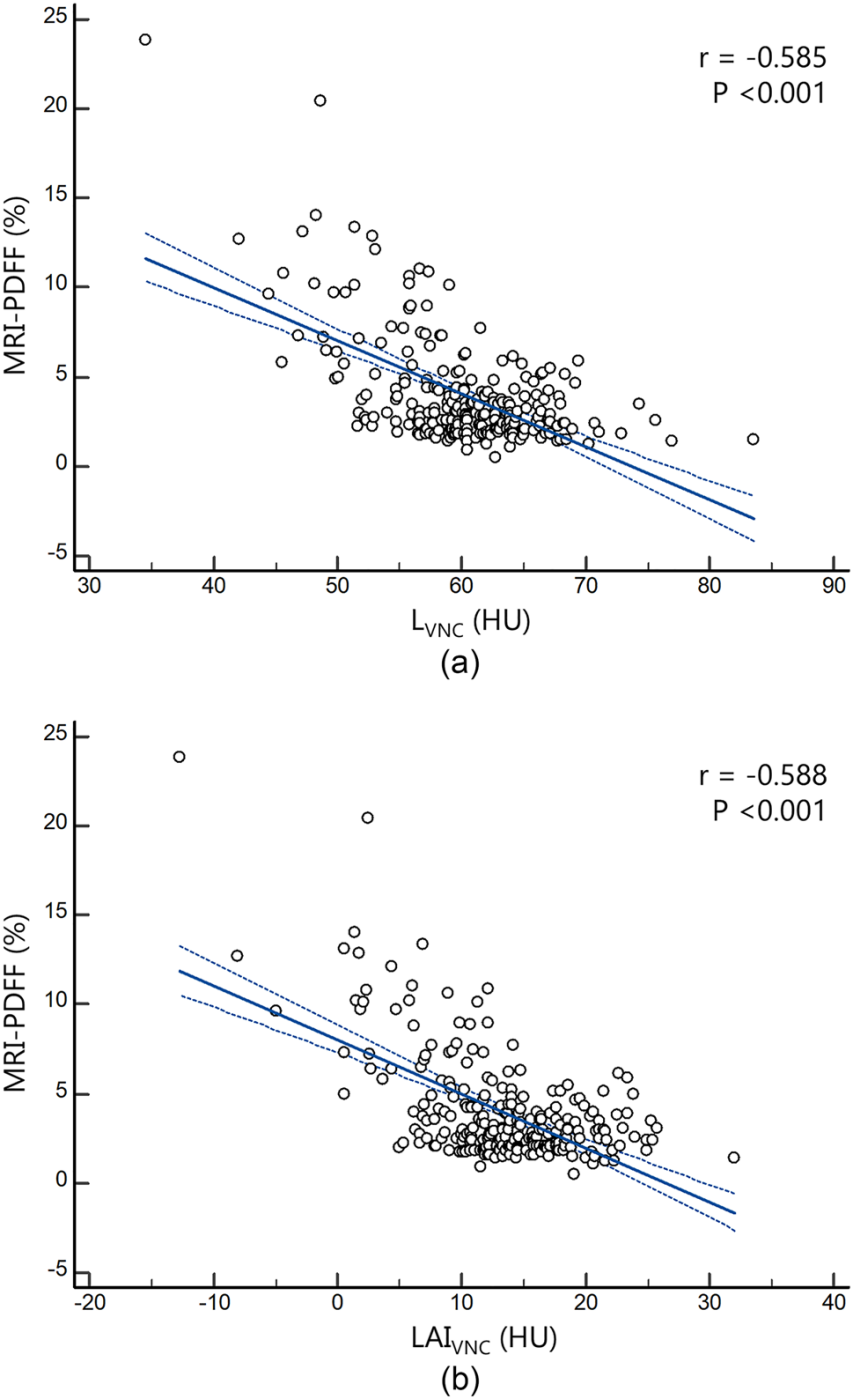
Scatter plots displaying the regression line (solid) and 95% confidence interval (dotted) of virtual non-contrast (VNC) parameters for hepatic steatosis (a: L_VNC_ and b: LAI_VNC_) with MRI-PDFF values. ‘r’ denotes Pearson’s correlation coefficient. L = volumetric mean CT attenuation values of the liver; S = volumetric mean CT attenuation values of the spleen; LAI = liver attenuation index, defined as L minus S; MRI-PDFF = magnetic resonance imaging-proton density fat fraction

Both L_VNC_ and LAI_VNC_ showed significant negative correlations with MRI-PDFF values (r = − 0.585 [95% CI − 0.661 ‒ − 0.498] and − 0.588 [95% CI − 0.663 ‒ − 0.500], respectively; Ps < 0.001) (Fig. 4).

### Diagnostic performance using MRI-PDFF as the reference standard

**Fig. 5:**
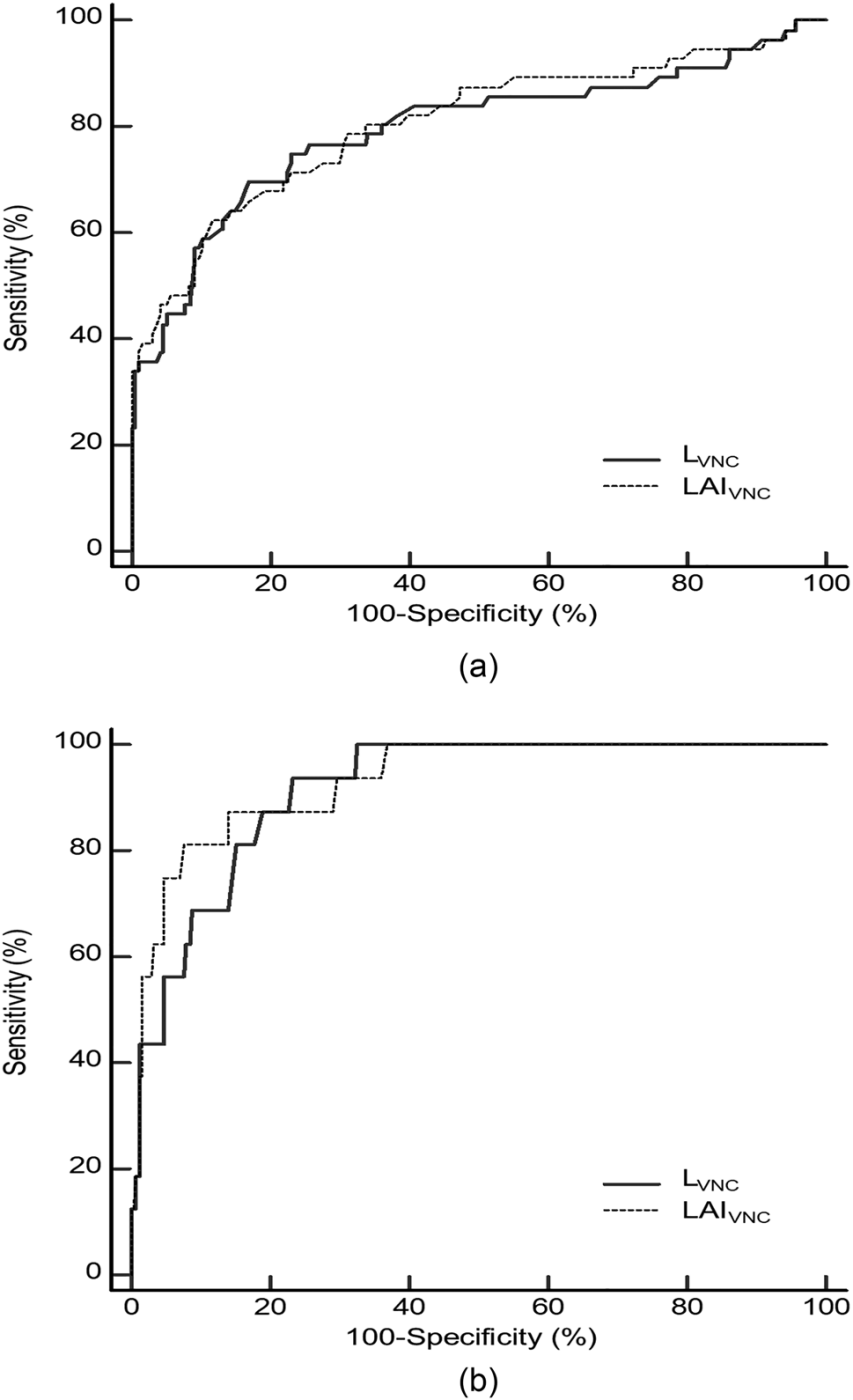
Graphs showing area under the receiver operating characteristic curve of virtual non-contrast (VNC) parameters for hepatic steatosis (L_VNC_ and LAI_VNC_) to identify mild steatosis (MRI-PDFF ≥ 5%) (**a**) and moderate-to-severe steatosis (MRI-PDFF ≥ 10%) (**b**). L = volumetric mean CT attenuation values of the liver; S = volumetric mean CT attenuation values of the spleen; LAI = liver attenuation index, defined as L minus S; MRI-PDFF = magnetic resonance imaging-proton density fat fraction

Table 2 summarizes the diagnostic performances of volumetric VNC parameters for hepatic steatosis, along with the cutoff values of each parameter for identifying MRI-PDFF ≥ 5% and ≥ 10%, respectively. L_VNC_ and LAI_VNC_ demonstrated AUCs of 0.795 and 0.806, respectively, for MRI-PDFF ≥ 5% (Fig. 5). For MRI-PDFF ≥ 10%, these parameters exhibited comparatively higher AUCs of 0.916 and 0.932, respectively (Fig. 5), when compared to the identification of MRI-PDFF ≥ 5%. L_VNC_ and LAI_VNC_ did not show significant differences in AUCs for both MRI-PDFF ≥ 5% and ≥ 10% (*P* = 0.626 and 0.328, respectively). The cutoff values of L_VNC_ and LAI_VNC_, aiming to achieve 80% sensitivity were 59.9 HU and 12.5 HU for MRI-PDFF ≥ 5%; and 55.9 HU and 6.9 HU for MRI-PDFF ≥ 10%, respectively (Table 2).

**Table 2:** Performance of virtual non-contrast CT parameters for assessing hepatic steatosis

Hepatic steatosis grades	VNC parameter	AUC (95% CI)	Cutoff (HU)		Sensitivity (%)	Specificity (%)
Mild steatosis (MRI-PDFF ≥ 5%)	L_VNC_	0.795 (0.740‒0.843)	Maximal Youden index	≤ 57.5	69.6 (39/56)	83.2 (163/196)
			> 80% sensitivity	≤ 59.9	80.4 (45/56)	63.8 (125/196)
			> 80% specificity	≤ 57.5	69.6 (39/56)	83.2 (163/196)
	LAI_VNC_	0.806 (0.751‒0.853)	Maximal Youden index	≤ 9.9	62.5 (35/56)	88.3 (173/196)
			> 80% sensitivity	≤ 12.5	80.4 (45/56)	66.3 (130/196)
			> 80% specificity	≤ 10.7	67.9 (38/56)	80.6 (158/196)
Moderate-to-severe steatosis (MRI-PDFF ≥ 10%)	L_VNC_	0.916 (0.874‒0.947)	Maximal Youden index	≤ 57.4	93.8 (15/16)	76.7 (181/236)
			> 80% sensitivity	≤ 55.9	81.3 (13/16)	84.8 (200/236)
			> 80% specificity	≤ 56.6	87.5 (14/16)	80.5 (190/236)
	LAI_VNC_	0.932 (0.893‒0.960)	Maximal Youden index	≤ 6.9	81.3 (13/16)	92.4 (218/236)
			> 80% sensitivity	≤ 6.9	81.3 (13/16)	92.4 (218/236)
			> 80% specificity	≤ 8.9	87.5 (14/16)	86.0 (203/236)

*VNC* virtual non-contrast, *AUC* area under the receiver operating characteristic curve, 95% *CI* 95% confidence interval, *MRI-PDFF* magnetic resonance imaging-proton density fat fraction, L = volumetric mean CT attenuation values of the liver, S = L = volumetric mean CT attenuation values of the spleen, *LAI* liver attenuation index, defined as L minus S. Unless otherwise specified, numbers in parentheses are used to calculate the percentages

When comparing the diagnostic performances of VNC and TNC parameters, L_VNC_ and LAI_VNC_ exhibited lower AUCs than those of L_TNC_ and LAI_TNC_ for MRI-PDFF ≥ 5% (AUCs, 0.795 vs. 0.825 and 0.806 vs. 0.866, respectively; Ps < 0.001). For MRI-PDFF ≥ 10%, L_VNC_ showed a lower AUC than L_TNC_ (0.916 vs. 0.948, *P* = 0.020), while the AUCs of LAI_VNC_ and LAI_TNC_ were not significantly different (0.932 vs. 0.964, *P* = 0.114).

## Discussion

This study focusing on VNC imaging generated from DECT scans, suggests that VNC parameters, derived from volumetric CT attenuation values of the liver and spleen obtained from automated organ segmentation, hold significant promise as a valuable method for hepatic steatosis assessment. Specifically, our study showed a significant correlation between VNC parameters such as L_VNC_ and LAI_VNC_ and hepatic fat content, as quantified using the gold standard MRI-PDFF. Additionally, these parameters demonstrated good diagnostic performance in assessing hepatic steatosis, with AUCs of approximately 0.8 for detecting mild steatosis (MRI-PDFF ≥ 5%), and over 0.9 for moderate steatosis (MRI-PDFF ≥ 10%). The implications of this study’s findings are substantial, particularly in expanding the scope of opportunistic CT screening for hepatic steatosis, given the increasing use of DECT in clinical practice. Furthermore, our study revealed robust agreement between volumetric CT attenuation values measured on VNC imaging of the liver and spleen and their respective manual ROI-based VNC measurements and volumetric TNC measurements, all achieving ICCs > 0.8. These findings underscore the reliability of automated volume-based VNC measurements, suggesting that a volume-based measurement approach has the potential to replace 2D-based measurement. In addition, in patients undergoing DECT, the study’s results indicate that hepatic steatosis assessment can utilize VNC imaging as a substitute for TNC, eliminating the need for additional TNC imaging acquisition.

Population-level screening for hepatic steatosis using CT has become a topic of considerable interest. Evaluations were predominantly conducted in non-contrast imaging settings to avoid confounding effects from contrast agents, utilizing indices based on liver attenuation values or corrected values, with the spleen as a reference. Previous studies employing automated volume-based measurements have highlighted the efficacy of CT attenuation-based parameters for hepatic steatosis in TNC, including L_TNC_ and LAI_TNC_, in large-scale cohorts [11, 27]. In the context of VNC application, reports suggest that manual 2D ROI-based parameters for hepatic steatosis, such as L_VNC_ and LAI_VNC_, exhibit a strong correlation with TNC parameters and show good diagnostic performance in identifying substantial hepatic steatosis [17, 28]. These findings align with our 3D-based VNC results, revealing strong correlations with TNC measurements and demonstrating satisfactory diagnostic performance for hepatic steatosis. However, in our study, while L_VNC_ showed good performance, it demonstrated relatively lower AUC values compared to TNC for both MRI-PDFF ≥ 5% and ≥ 10%. This may be attributed to inconsistent and suboptimal iodine extraction during VNC generation from contrast-enhanced DECT imaging, affecting the CT attenuation values of the liver on VNC, thereby potentially interfering with the prediction of hepatic steatosis based on CT attenuation values. For LAI, although its performance for hepatic steatosis assessment was also slightly lower in VNC compared to TNC, statistically significant differences were not observed for MRI-PDFF ≥ 10%. When comparing LAI_VNC_ to L_VNC_, LAI_VNC_ tended to perform slightly better in hepatic steatosis assessment. This trend is likely due to the corrective effect of using the spleen as a reference, which was also reported in prior 2D-based VNC studies [17, 29, 30] as well as TNC studies [22, 31].

Our study demonstrated that the AUCs of VNC parameters in identifying MRI-PDFF ≥ 10% were superior compared to those of MRI-PDFF ≥ 5%. This result can be explained by the limited sensitivity of CT in detecting mild steatosis [32, 33]. The study’s findings suggest cutoff values for L_VNC_ and LAI_VNC_ under specific conditions, aiming to provide a maximal Youden index, > 80% sensitivity, and > 80% specificity, which could aid in interpreting the measured values. However, it is important to note that the performance of these cutoff values has not been externally validated. According to a recent study introducing a CT-MRI conversion equation applicable to TNC imaging performed at 120 kVp, the CT attenuation values of the liver equivalent to MRI-PDFF of 5% and 10% are 57.2 HU and 48.6 HU, respectively [23]. However, considering that CT attenuation values may be affected by the CT scanner type and kVp setting [34], and taking into account the small but existing systematic differences in measurements between VNC and TNC, as shown in our study, further research is necessary to determine appropriate cutoff values for VNC application.

In our study, volumetric VNC measurements demonstrated strong agreement with manual ROI-based measurements, exhibiting high ICC values consistent with the findings of a previous TNC study of the liver [11]. However, our study also showed that the volumetric measurements were slightly lower compared to the manual ROI-based measurements, with mean biases of − 5.1 HU and − 5.5 HU for liver and spleen, respectively. This difference may be related to the partial inclusion of intrahepatic vasculature when using the automated volumetric approach, resulting in a trend of decreasing HU values, particularly when the liver parenchymal HU exceeds the blood pool HU [11]. Unlike the ROI placement method, which selects relatively homogeneous areas of the liver, the volumetric approach involves whole-liver segmentation, encompassing the subcapsular peripheral hepatic parenchyma and areas near the falciform ligament and hilum, which frequently contain greater fat deposits [35]. Given the observed differences in the measurement values, the direct adoption of 2D-based cutoffs for volume-based methods may not be deemed optimal. Consequently, the establishment of cutoff values tailored to 3D methods and their subsequent applications could be considered a more appropriate approach.

This study has several limitations. First, our study population consisted of a relatively small number of potential liver donor candidates who were generally healthy and young and none had severe hepatic steatosis. The selection of this population was driven by the availability of both CT and MRI-PDFF values at our institution, which allowed for the evaluation of CT parameters against a gold standard. However, inherent selection bias in our patient population resulting from this choice may have limited the generalizability of our study results. Further prospective studies involving large populations with varying degrees of hepatic steatosis, as well as broader spectrum of underlying liver diseases, are required to validate and enhance the generalizability of our findings. Second, VNC images in our study were generated using DECT-PVP imaging. A previous study indicated that the attenuation values of abdominal organs might vary depending on the derived imaging phase [36]. Thus, our results, especially the cut-off values, may not be directly applicable to VNC images derived from arterial or delayed phases. However, addressing these limitations is anticipated to be possible with future advancements in the technical feasibility of achieving accurate iodine extraction during the VNC generation process.

In conclusion, volumetric CT attenuation-based parameters from VNC images generated by DECT acquired through automated 3D segmentation of the liver and spleen have the potential for opportunistic screening of hepatic steatosis, as an alternative to TNC images. While promising, it's important to acknowledge the limitations outlined in our study, such as the relatively small and selective study population and the potential variation in attenuation values depending on the derived imaging phase for VNC generation. Addressing these limitations and further advancements in the technical feasibility of VNC generation may pave the way for enhanced applicability of this method in clinical practice.

### Supplementary Information

Below is the link to the electronic supplementary material.

Supplementary file 1 (DOCX 188 kb)

## Data Availability

The datasets used during the current study are available from the corresponding author on reasonable request.

## References

[1] Starekova J, Hernando D, Pickhardt PJ, Reeder SB (2021). Quantification of liver fat content with CT and MRI: state of the art. Radiology.

[2] Ma X, Holalkere NS, Kambadakone RA, Mino-Kenudson M, Hahn PF, Sahani DV (2009). Imaging-based quantification of hepatic fat: methods and clinical applications. Radiographics.

[3] Anstee QM, Targher G, Day CP (2013). Progression of NAFLD to diabetes mellitus, cardiovascular disease or cirrhosis. Nat Rev Gastroenterol Hepatol.

[4] Pais R, Barritt AS, Calmus Y, Scatton O, Runge T, Lebray P, Poynard T, Ratziu V, Conti F (2016). NAFLD and liver transplantation: current burden and expected challenges. J Hepatol.

[5] Tapper EB, Lok AS (2017). Use of liver imaging and biopsy in clinical practice. N Engl J Med.

[6] Machado MV, Cortez-Pinto H (2013). Non-invasive diagnosis of non-alcoholic fatty liver disease. A critical appraisal. J Hepatol.

[7] Ringe KI, Yoon JH (2023). Strategies and techniques for liver magnetic resonance imaging: new and pending applications for routine clinical practice. Korean J Radiol.

[8] Boyce CJ, Pickhardt PJ, Kim DH, Taylor AJ, Winter TC, Bruce RJ, Lindstrom MJ, Hinshaw JL (2010). Hepatic steatosis (fatty liver disease) in asymptomatic adults identified by unenhanced low-dose CT. AJR Am J Roentgenol.

[9] Park HJ, Park B, Lee SS (2020). Radiomics and deep learning: hepatic applications. Korean J Radiol.

[10] Wang K, Mamidipalli A, Retson T, Bahrami N, Hasenstab K, Blansit K, Bass E, Delgado T, Cunha G, Middleton MS, Loomba R, Neuschwander-Tetri BA, Sirlin CB, Hsiao A (2019). Automated CT and MRI liver segmentation and biometry using a generalized convolutional neural network. Radiol Artif Intell.

[11] Graffy PM, Sandfort V, Summers RM, Pickhardt PJ (2019). Automated liver fat quantification at nonenhanced abdominal CT for population-based steatosis assessment. Radiology.

[12] Pickhardt PJ, Blake GM, Graffy PM, Sandfort V, Elton DC, Perez AA, Summers RM (2021). Liver steatosis categorization on contrast-enhanced CT using a fully automated deep learning volumetric segmentation tool: evaluation in 1204 healthy adults using unenhanced CT as a reference standard. AJR Am J Roentgenol.

[13] So A, Nicolaou S (2021). Spectral computed tomography: fundamental principles and recent developments. Korean J Radiol.

[14] Grant KL, Flohr TG, Krauss B, Sedlmair M, Thomas C, Schmidt B (2014). Assessment of an advanced image-based technique to calculate virtual monoenergetic computed tomographic images from a dual-energy examination to improve contrast-to-noise ratio in examinations using iodinated contrast media. Invest Radiol.

[15] Bae JS, Lee DH, Joo I, Jeon SK, Han JK (2019). Utilization of virtual non-contrast images derived from dual-energy CT in evaluation of biliary stone disease: virtual non-contrast image can replace true non-contrast image regarding biliary stone detection. Eur J Radiol.

[16] Sauter AP, Muenzel D, Dangelmaier J, Braren R, Pfeiffer F, Rummeny EJ, Noël PB, Fingerle AA (2018). Dual-layer spectral computed tomography: virtual non-contrast in comparison to true non-contrast images. Eur J Radiol.

[17] Kang HJ, Lee DH, Park SJ, Han JK (2021). Virtual noncontrast images derived from dual-energy CT for assessment of hepatic steatosis in living liver donors. Eur J Radiol.

[18] Guo Z, Blake GM, Li K, Liang W, Zhang W, Zhang Y, Xu L, Wang L, Brown JK, Cheng X, Pickhardt PJ (2020). Liver fat content measurement with quantitative CT validated against MRI proton density fat fraction: a prospective study of 400 healthy volunteers. Radiology.

[19] Yoon SB, Lee IS, Choi MH, Lee K, Ham H, Oh HJ, Park SH, Lim CH, Choi MG (2017). Impact of fatty liver on acute pancreatitis severity. Gastroenterol Res Pract.

[20] Hokkanen A, Hämäläinen H, Laitinen TM, Laitinen TP (2021). Test-retest reliability of the assessment of fatty liver disease using low-dose computed tomography in cardiac patients. Front Med (Lausanne).

[21] Lee SW, Park SH, Kim KW, Choi EK, Shin YM, Kim PN, Lee KH, Yu ES, Hwang S, Lee SG (2007). Unenhanced CT for assessment of macrovesicular hepatic steatosis in living liver donors: comparison of visual grading with liver attenuation index. Radiology.

[22] Park SH, Kim PN, Kim KW, Lee SW, Yoon SE, Park SW, Ha HK, Lee MG, Hwang S, Lee SG, Yu ES, Cho EY (2006). Macrovesicular hepatic steatosis in living liver donors: use of CT for quantitative and qualitative assessment. Radiology.

[23] Pickhardt PJ, Graffy PM, Reeder SB, Hernando D, Li K (2018). Quantification of Liver fat content with unenhanced MDCT: phantom and clinical correlation with MRI proton density fat fraction. AJR Am J Roentgenol.

[24] Campo CA, Hernando D, Schubert T, Bookwalter CA, Pay AJV, Reeder SB (2017). Standardized approach for ROI-based measurements of proton density fat fraction and R2* in the liver. AJR Am J Roentgenol.

[25] Koo TK, Li MY (2016). A guideline of selecting and reporting intraclass correlation coefficients for reliability research. J Chiropr Med.

[26] Guilford JP (1950) Fundamental statistics in psychology and education. McGraw-Hill, New York

[27] Jirapatnakul A, Reeves AP, Lewis S, Chen X, Ma T, Yip R, Chin X, Liu S, Perumalswami PV, Yankelevitz DF, Crane M, Branch AD, Henschke CI (2020). Automated measurement of liver attenuation to identify moderate-to-severe hepatic steatosis from chest CT scans. Eur J Radiol.

[28] Kaza RK, Raff EA, Davenport MS, Khalatbari S (2017). Variability of CT attenuation measurements in virtual unenhanced images generated using multimaterial decomposition from fast kilovoltage-switching dual-energy CT. Acad Radiol.

[29] Borhani AA, Kulzer M, Iranpour N, Ghodadra A, Sparrow M, Furlan A, Tublin ME (2017). Comparison of true unenhanced and virtual unenhanced (VUE) attenuation values in abdominopelvic single-source rapid kilovoltage-switching spectral CT. Abdom Radiol (NY).

[30] Slebocki K, Kraus B, Chang DH, Hellmich M, Maintz D, Bangard C (2017). Incidental findings in abdominal dual-energy computed tomography: correlation between true noncontrast and virtual noncontrast images considering renal and liver cysts and adrenal masses. J Comput Assist Tomogr.

[31] Kim DY, Park SH, Lee SS, Kim HJ, Kim SY, Kim MY, Lee Y, Kim TK, Khalili K, Bae MH, Lee JY, Lee SG, Yu ES (2010). Contrast-enhanced computed tomography for the diagnosis of fatty liver: prospective study with same-day biopsy used as the reference standard. Eur Radiol.

[32] Bohte AE, van Werven JR, Bipat S, Stoker J (2011). The diagnostic accuracy of US, CT, MRI and 1H-MRS for the evaluation of hepatic steatosis compared with liver biopsy: a meta-analysis. Eur Radiol.

[33] Pickhardt PJ, Park SH, Hahn L, Lee SG, Bae KT, Yu ES (2012). Specificity of unenhanced CT for non-invasive diagnosis of hepatic steatosis: implications for the investigation of the natural history of incidental steatosis. Eur Radiol.

[34] Cropp RJ, Seslija P, Tso D, Thakur Y (2013). Scanner and kVp dependence of measured CT numbers in the ACR CT phantom. J Appl Clin Med Phys.

[35] Martí-Aguado D, Jiménez-Pastor A, Alberich-Bayarri Á, Rodríguez-Ortega A, Alfaro-Cervello C, Mestre-Alagarda C, Bauza M, Gallén-Peris A, Valero-Pérez E, Ballester MP, Gimeno-Torres M, Pérez-Girbés A, Benlloch S, Pérez-Rojas J, Puglia V, Ferrández A, Aguilera V, Escudero-García D, Serra MA, Martí-Bonmatí L (2022). Automated whole-liver MRI segmentation to assess steatosis and iron quantification in chronic liver disease. Radiology.

[36] Kim S, Kang BS, Kwon WJ, Bang M, Lim S, Park GM, Lee TY (2020). Abdominal organs attenuation values and abdominal aortic calcifications on virtual and true noncontrast images obtained with third-generation dual-source dual-energy computed tomography. J Comput Assist Tomogr.

