# Supplementary material for: Automated hepatic steatosis assessment on dual-energy CT-derived virtual non-contrast images through fully-automated 3D organ segmentation
Source: Radiol Med. 2024 Jun 13;129(7):967–76. doi: 10.1007/s11547-024-01833-8 (PMC11252222; doi:10.1007/s11547-024-01833-8)

**Supplementary 1. Detailed CT protocol**

The CT protocols for liver donor candidates at our institution consisted of true non-contrast (TNC), arterial phase (AP), portal venous phase (PVP), and delayed phase (DP) images. Post-contrast images, i.e., AP, PVP, and DP images, were obtained in the dual-energy CT mode using 80 kVp and 250 mAs for the A-tube set, and 150 kVp and 125 mAs for the B-tube set, while TNC images were obtained in the single-energy CT mode at 120 kVp and 180 mAs. The tube currents were adjusted in real-time by automatic dose modulation provided by the manufacturer (Care Dose 4D; Siemens Healthineers) to maintain the optimal level of image noise. The detailed CT parameters were as follows: collimation, 192 x 0.6 mm; rotation time, 0.5 second; pitch, 0.6; slice thickness, 2 mm; reconstruction interval, 1 mm; and kernel Br40. For post-contrast imaging, iobitridol (Xenetix 350; Guerbet) was administered at a dose of 520 mg/kg body weight using a power injector (Stellent D, Medrad) over 30 seconds at a rate of 2–5 mL/s, followed by a 20–30-mL saline flush. The AP scan commenced automatically 17 seconds after the abdominal aorta reached 80 HU at 150 kVp using the care bolus technique. PVP and DP images were acquired 70 and 180 seconds after contrast media administration, respectively. The PVP scan covered the entire abdomen, from the top of the higher hemi-diaphragmatic dome to the level of the pelvic inlet, while the other phases included the entire liver.

**Supplementary Figure 1.** Bland-Altman plots of volumetric versus 2D region-of-interest measurements of CT attenuation values of the liver (a) and spleen (b) on virtual non-contrast imaging.

*ROI = region-of-interest, VNC = virtual non-contrast, L = volumetric mean CT attenuation values of the liver; S = volumetric mean CT attenuation values of the spleen.*


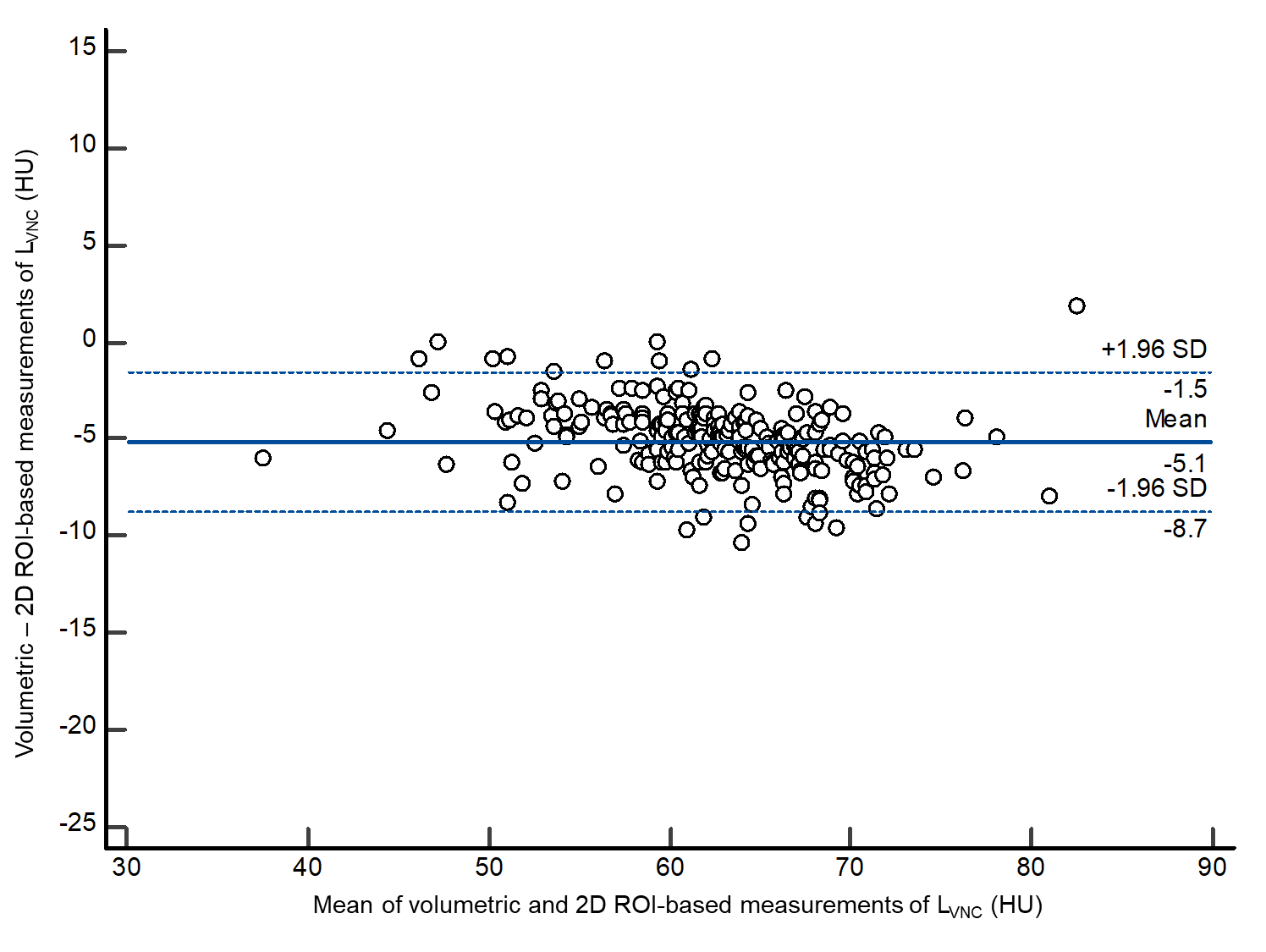
(a)

(b)


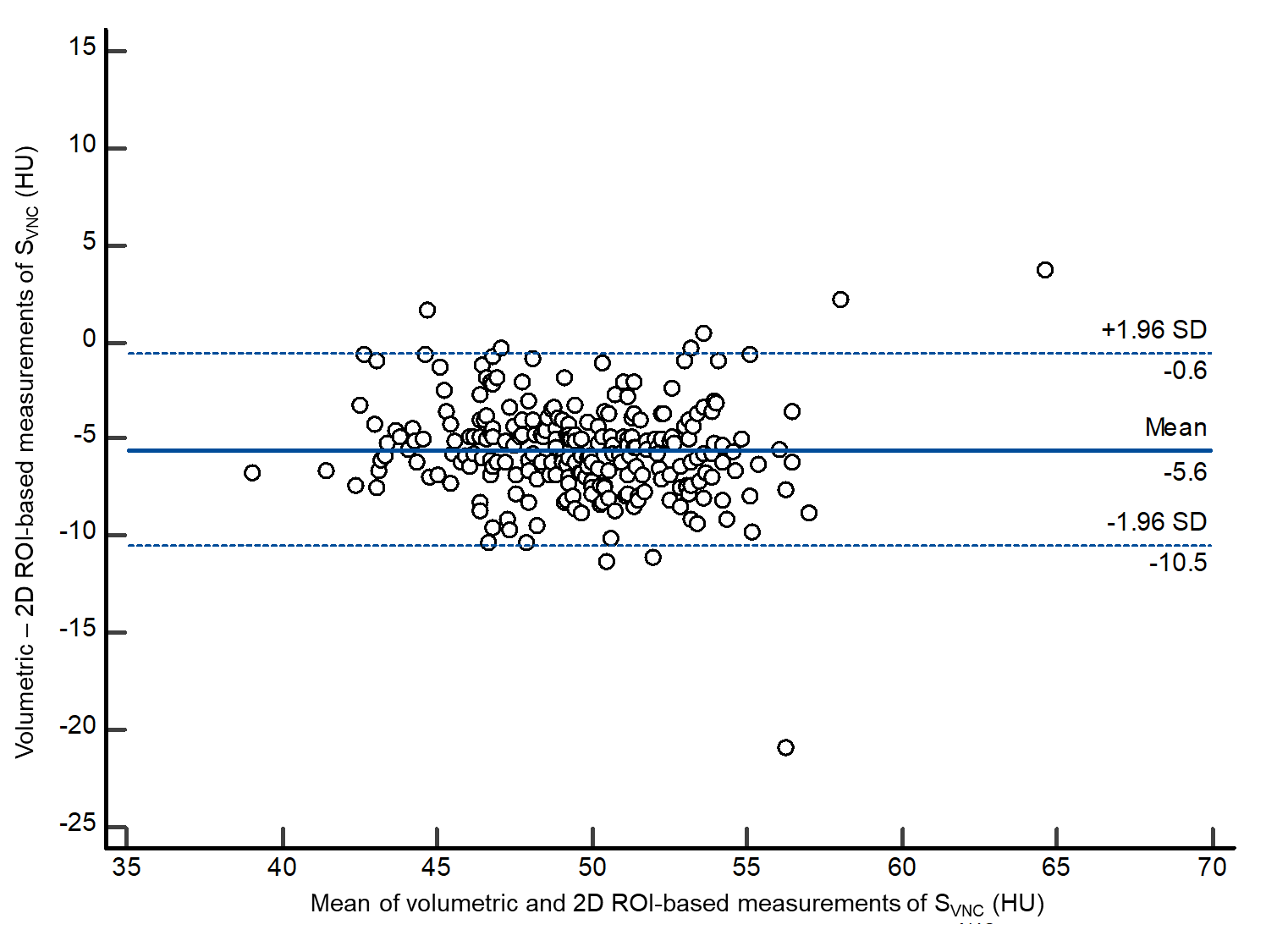


**Supplementary Figure 2.** Bland-Altman plots of virtual non-contrast versus true non-contrast imaging for the measurement of volumetric mean CT attenuation values of the liver (a) and spleen (b).

*VNC = virtual non-contrast, TNC = true non-contrast, L = volumetric mean CT attenuation values of the liver; S = volumetric mean CT attenuation values of the spleen*

(a)


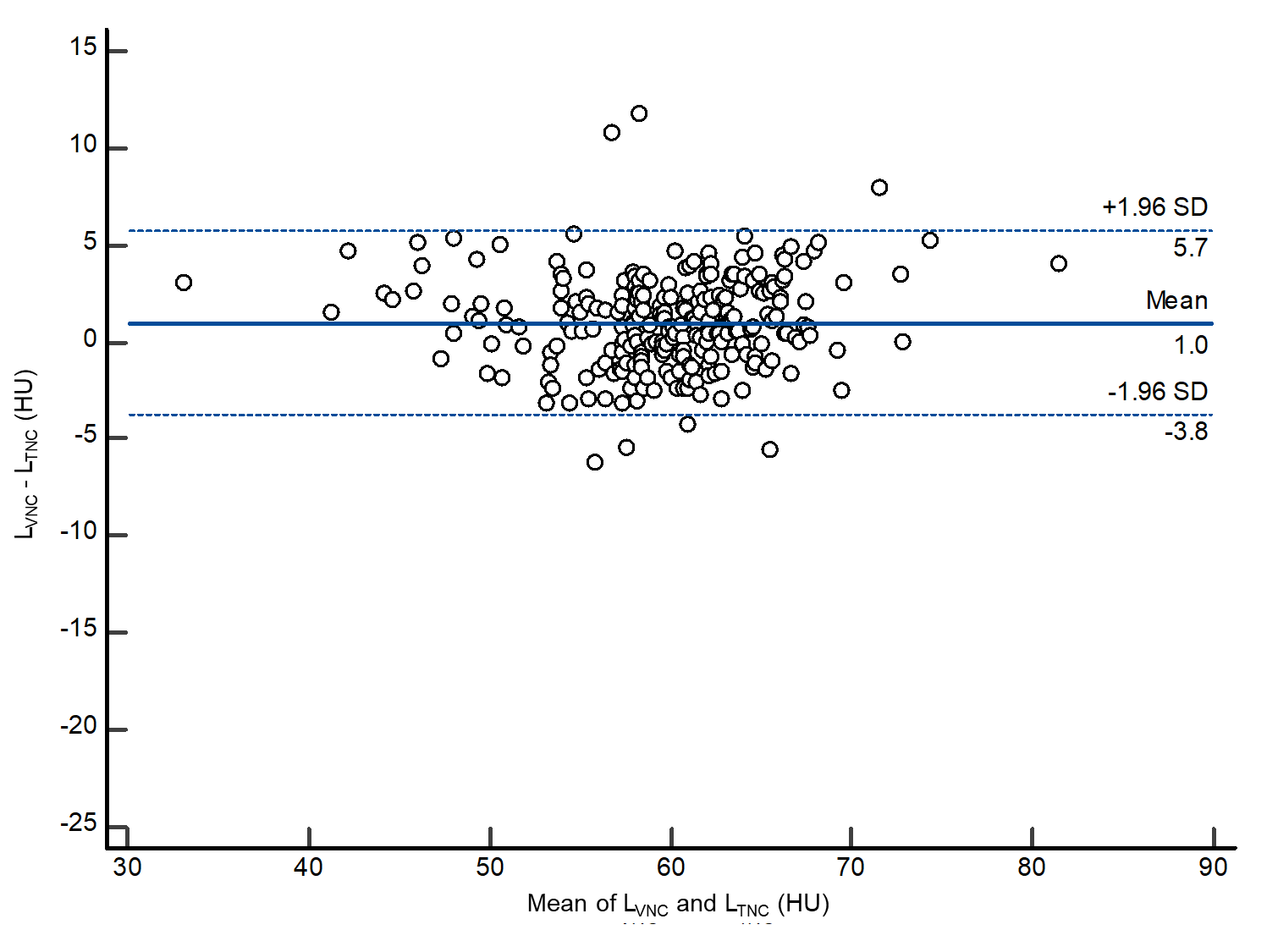


(b)


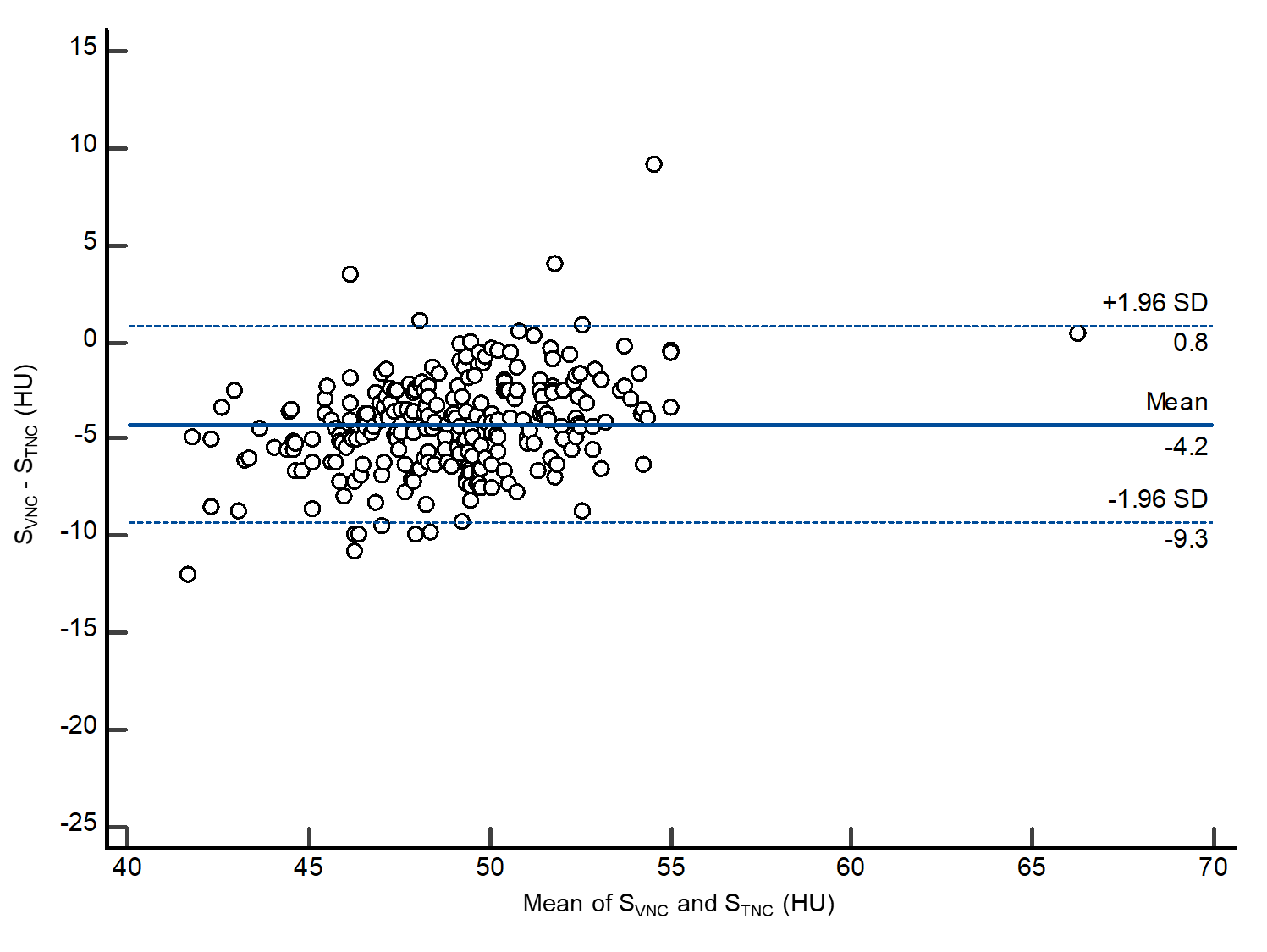

Supplement: Supplementary file 1 — (DOCX 188 kb) [file 11547_2024_1833_MOESM1_ESM.docx]
